# Kidney Biopsy in Patients with Cancer along the Last Decade: A Multicenter Study

**DOI:** 10.3390/jcm11102915

**Published:** 2022-05-21

**Authors:** Mónica Bolufer, Clara García-Carro, Miquel Blasco, Luis F. Quintana, Amir Shabaka, Cristina Rabasco, Juliana Draibe, Ana Merino, María Rosa Melero, Fabiola Alonso, Anna Buxeda, Paula Batalha, Maria Teresa Visús, Maria José Soler

**Affiliations:** 1 Nephrology Department, Vall d’Hebron University Hospital, Vall d’Hebron Institute of Research, CSUR National Unit of Expertise for Complex Glomerular Diseases of Spain, 08035 Barcelona, Spain; mbolufer@vhebron.net; 2 Nephrology Department, San Carlos Clinical University Hospital, 28034 Madrid, Spain; cgcarro@salud.madrid.org; 3 Department of Nephrology and Renal Transplantation, Hospital Clínic, IDIBAPS, CSUR National Unit of Expertise for Complex Glomerular Diseases of Spain, 08035 Barcelona, Spain; miblasco@clinic.cat (M.B.); lfquinta@clinic.cat (L.F.Q.); 4 Nephrology Department, Fundación Alcorcón, 28922 Alcorcón, Spain; amirshabaka@hotmail.com; 5 Nephrology Department, Córdoba Hospital, 14071 Córdoba, Spain; cristina.rabasco.sspa@juntadeandalucia.es; 6 Nephrology Department, Bellvitge Hospital, 08907 Hospitalet Llobregat, Spain; jbordignon@bellvitgehospital.cat; 7 Nephrology Department, Dr. Josep Trueta Hospital, 17007 Gerona, Spain; anamerinoribas@gmail.com; 8 Nephrology Department, Gregorio Marañón Hospital, 28007 Madrid, Spain; mrosamelero@yahoo.es; 9 Nephrology Department, Virgen Macarena Hospital, 41009 Sevilla, Spain; fabiolaalonsogarcia@gmail.com; 10 Nephrology Department, Hospital del Mar, 08003 Barcelona, Spain; abuxeda@psmar.cat; 11 Nephrology Department, Virgen del Rocío Hospital, 41013 Sevilla, Spain; pbatalhac@gmail.com; 12 Nephrology Department, Navarra Hospital, 31008 Pamplona, Spain; mt.visus.fernandezdemanzanos@navarra.es

**Keywords:** onconephrology, renal pathology, kidney biopsy, solid organ neoplasm

## Abstract

Background: Currently, following the new advances in cancer treatments and the increasing prevalence of kidney disease in the population, more kidney biopsies are being performed. The aim of our study is to analyze clinical and histological characteristics of patients with active solid organ malignancy who underwent kidney biopsy. This is a multi-center collaborative retrospective study supported by groups GLOSEN/Onconephrology from the Spanish Society of Nephrology. Clinical, demographical and histological data were collected. Results: A total of 148 patients with cancer who underwent a kidney biopsy from 12 hospitals were included. 64.3% men and mean age of 66.9 years old. The indications for biopsy were acute renal injury (67.1%), proteinuria (17.1%), exacerbated chronic kidney disease (8.2%), and chronic kidney disease (7.5%). Most frequent malignances were lung (29.1%) and abdominal (25%), with 49.7% metastatic cancer. As oncospecific treatment, 28% received chemotherapy, 29.3% immunotherapy, 19.3% specific therapies, and 2.1% conservative treatment. At the time of kidney biopsy, median creatinine was of 2.58 mg/dL [1.81–4.1 (IQ 25–75)], median urine protein-to-creatinine ratio of 700 mg/g [256–2463 (IQ 25–75)] and 53.1% presented hematuria. The most frequent renal biopsy diagnoses were: acute interstitial nephritis (39.9%), acute tubular necrosis (8.8%), IgA nephropathy (7.4%) and membranous nephropathy (6.1%). Median follow-up was 15.2 months [5.7–31.4 (IQ 25–75)]. Conclusions: There is a new trend in kidney disease and cancer patients in terms of diagnosis and treatment. Acute interstitial nephritis has established itself as the most common kidney injury in patients with cancer who underwent a kidney biopsy. Renal biopsy is a valuable tool for diagnosis, treatment, and prognosis of solid organ cancer patients with kidney damage.

## 1. Introduction

The relationship between cancer and kidney disease is bidirectional, which increases mortality and morbidity in a pool of high complexity patients [1]. Recently, the cancer paradigm has changed. The advances in cancer prevention, knowledge of the immunological biology of tumors, and new treatment possibilities have led to an improvement in the life expectancy of patients with cancer [2]. However, these new oncological treatments are often linked to adverse renal events that require nephrological evaluation [3,4,5].

At a onconephrology clinic, nephrologists must usually answer the question about the etiology of the renal event in the patient with cancer. The cancer itself could be responsible for the renal disease. However, the oncospecific treatment could also be related to the kidney injury. Furthermore, sometimes, the nephrologist, depending on the renal diagnostic suspicion, should recommend the discontinuation (or not) of the oncologic drugs.

A total of 15–17% of patients who are treated with checkpoint inhibitors develop acute kidney injury [6,7], mainly secondary to immune-mediated acute nephritis [8,9,10,11,12] but also associated with glomerular damage [13]. The presence of AKI (Acute Kidney Injury) in these patients has been associated with increased mortality risk [7]. This renal damage induced by new treatments is frequently treatable, and kidney function can be recovered [14]. For these reasons, the study of specific clinical and histologic patterns is needed to guide more specific therapies and to reach better outcomes.

The recommendations for kidney biopsy in patients with cancer have been recently updated [15]. Currently, kidney biopsy is recommended in those who present with new-onset proteinuria ≥1 g per day or worsening renal function when the diagnosis of kidney disease cannot be otherwise established. Furthermore, the indication for kidney biopsy in patients with cancer and a good prognosis should be similar to the general population. To our knowledge, in previous studies, few series reporting kidney biopsies in patients with advanced cancer have been published; thus, it is difficult to draw conclusions from the available information.

Collecting retrospective data on histologic patterns and clinical evolution of patients with cancer who underwent kidney biopsy could help the nephrology community to understand the change in kidney damage linked to the transformation of oncology that is happening nowadays [16]. The aim of our study is to explore the clinical and renal histologic characteristics of patients with cancer who underwent a kidney biopsy in Spain in the last decade, as well as treatments and their renal and general outcomes.

## 2. Materials and Methods

### 2.1. Sample Selection

We conducted a retrospective multicenter observational study of patients with solid organ neoplasia who underwent a renal biopsy of the native kidney in Spain between January 2010 and March 2021. Patients were followed up until May 2021. All of the patients older than 18 years old who underwent a kidney biopsy while presenting a solid organ malignancy were eligible except those with a previous kidney transplant. Biopsies performed the year before the diagnosis of neoplasia were included. We included patients from nephrology departments belonging to the Spanish Glomerular Study Group and/or Spanish Onconephrology Study Group. The Ethical Committee of Vall d’Hebron University Hospital approved the study protocol (PR(AG)260/2019).

### 2.2. Clinical Variables

Clinical and laboratory data were evaluated at the time of kidney biopsy. Demographic and clinical data, including age, sex, ethnicity, high blood pressure, diabetes mellitus, cardiovascular disease, and regular chronic medications, were collected. Oncological disease characteristics were also recorded, as well as laboratory parameters. Baseline creatinine was defined as the last measurement before the renal event that motivated the biopsy. AKI was defined according to the Kidney Disease Improving Global Outcomes (KDIGO) criteria [17].

### 2.3. Statistical Analysis

The data were analyzed using IBM SPSS Statistics Version 25.0. IBM Corp., Armonk, NY, USA. The Kolmogorov–Smirnov test was applied to determine whether quantitative variables were normally distributed. The results were expressed as frequencies for categorical variables and as mean ± standard deviation (SD), or median and interquartile range (IQR), for continuous variables. The comparison of continuous variables between two groups was performed by either a Student’s T or Mann–Whitney U, depending on the distribution of the variable. A Cox survival analysis adjusted for clinical conditions was performed to identify risk factors associated with mortality. A two-sided *p*-value < 0.05 was considered statistically significant.

## 3. Results

### 3.1. Baseline Chracteristics of Population

**Figure 1:**
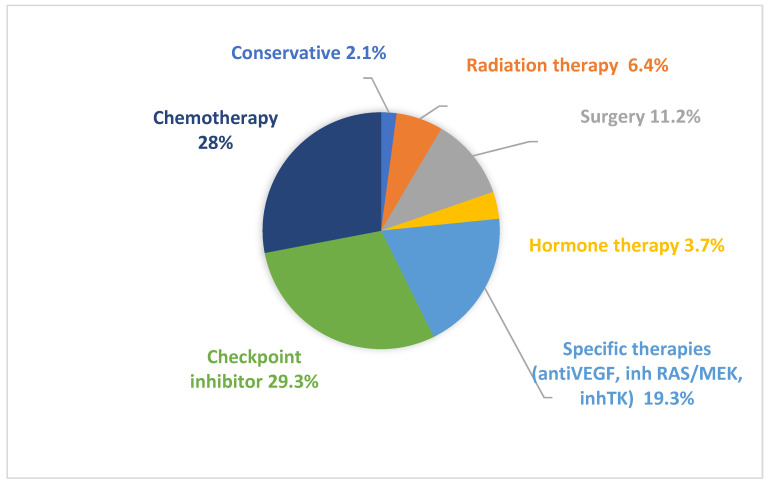
Oncospecific treatment prior to kidney biopsy. Types of treatments received by patients before undergoing kidney biopsy.

A total of 148 patients from 12 Spanish hospitals were included in the study (baseline characteristics three months before renal biopsy 99.5 days [40.50–215.75 (IQ 25–75)] as shown in Table 1 and Table S1). The mean age was 66.9 years old at the time of biopsy, and 64.2% were men, 29.7% had diabetes, 62.2% had high blood pressure, 12.2% were under non-steroidal anti-inflammatory drugs treatment, and 62.8% were receiving renin-angiotensin system blockers. The median baseline creatinine was 1 mg/dL, and 15.5% of patients presented baseline creatinine > 1.5 mg/dL. The most frequent malignancies were lung (29.1%), abdominal (25%), genitourinary (19.6%), and melanoma (10.8%). Overall, 49.7% of patients had metastatic disease at the moment of kidney biopsy. The oncospecific treatment prior to kidney biopsy is summarized in Figure 1.

### 3.2. Administered Therapies

Briefly, the most frequently used therapies were chemotherapy (28%), immunotherapy (29.3%), and specific targeted therapies (19.3%). Overall, 54 patients included were treated with checkpoint inhibitors (CPI), and in 22.3% of them, two CPI drugs were administered. At the time of kidney biopsy, median creatinine was 2.58 mg/dL (21.8 mL/min/1.73 m^2^ glomerular filtration rate) and the median urine protein/creatinine ratio was 700 mg/g, while 23% of patients presented with nephrotic-range proteinuria. Furthermore, 53.1% presented hematuria, 10.8% eosinophiluria, and 6.8% hemolytic anemia and/or low platelet count. Autoimmunity workup revealed that 6.8% of patients presented positivity for antineutrophil cytoplasmic antibodies and 10.1% showed decreased serum levels of C3 and/or C4; these data are summarized in Table 2.

### 3.3. Renal Biopsy Diagnosis

After evaluation by nephrology, the most frequent indication for renal biopsy was acute renal failure, in 67.1% of cases; 51% presented with AKI 1, 17% AKI 2, and 31% AKI 3. Renal biopsy was indicated in 7.5% of patients with stage G3a and G3b chronic kidney disease, while exacerbated chronic kidney disease led to histopathological analysis in 8.2%. Proteinuria was the indication for renal biopsy in 17.1%, and three patients presented with nephrotic syndrome.

The mean time between the diagnosis of neoplasia and renal biopsy was of 1 year [0.6–2.4 (IQ 25–75)]. The diagnosis of renal disease preceded the diagnosis of neoplasia in only six patients. The most frequent kidney histological diagnosis was acute interstitial nephritis (AIN) (39.9%), followed by acute tubular necrosis (8.8%), IgA nephropathy (7.4%), membranous nephropathy (6.1%), and thrombotic microangiopathy (5.4%) (Table 3). Acute interstitial nephritis was more frequently observed in the period from 2017 to 2021 as compared with the period from 2010 to 2016 (*p* = 0.001) (Supplementary Figure S1).

The nephrologist’s opinion about the etiology of the renal event was recorded. According to this, 56% of the diagnoses were secondary to the antineoplastic treatment that the patients were receiving at the time of the kidney biopsy. The most frequently responsible drugs were: immunotherapy (CTLA4 + PD1/PD1/PD-L1), MEK/B RAF inhibitor, and tyrosine kinase inhibitor in patients who developed biopsy-proven AIN. Acute tubular necrosis was related to oxaliplatin, alectinib, and capecitabine, while VEGF (Vascular Endothelial Growth Factor) was responsible for thrombotic microangiopathy.

A total of 13% of the renal events were secondary to the cancer itself, highlighting several cases: dominant IgA post-infectious glomerulonephritis after abscess secondary to resection of pulmonary metastasis, membranous nephropathy in the case of bladder and anal epidermoid neoplasia, amyloidosis in intestinal cancer, as well as diverse extracapillary glomerulonephritis and thrombotic microangiopathy secondary to gastrointestinal cancer.

Finally, in 31% of patients, kidney pathology was not related to cancer or its treatment. (Table 4 and Supplementary Figure S2).

There were 59 patients diagnosed with AIN: 66% (*n* = 39) was secondary to treatment with immunotherapy, and the remaining 34% (*n* = 20) was mainly due to antibiotics and other anticancer drugs, as detailed in Table 5 and Supplementary Figure S2. Interestingly, 15 patients received immunotherapy, but the renal biopsy revealed another pathology different from AIN: nephroangiosclerosis, acute tubular necrosis, membranous nephropathy, endocapillary, and extracapillary glomerulonephritis, IgA nephropathy, thrombotic microangiopathy, and amyloidosis.

**Figure 2:**
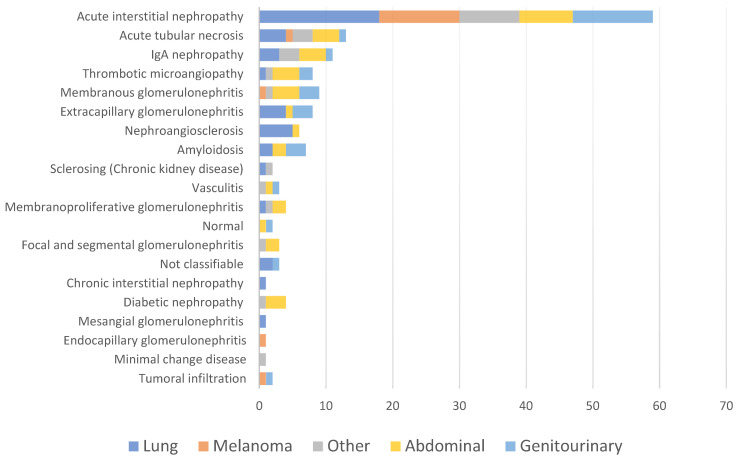
Pathological diagnosis based on the type of cancer. Classification of renal histological diagnoses in relation to the solid organ neoplasm of the patient.

We did not find any relation between the renal diagnosis and the type of neoplasia (Figure 2).

After kidney biopsy, 104 patients out of the 148 were specifically treated according to the renal diagnosis. A total of 59 (100%) patients with acute interstitial nephritis were treated with steroids; 21 (35.6%) of them received intravenous methylprednisolone pulses. In two cases, mycophenolate mofetil was also administered. The median duration of steroids treatment was 3.7 [1.8–9.3 (IQ 25–75)] months. The subanalysis of the 52 cases of AIN secondary to oncological treatment evidenced that 21% (*n* = 11) relapsed at a median time of 3.6 months ([2.4–5.2 (IQ 25–75)]. In more than half (*n* = 6), the recurrence occurred within the corticosteroid withdrawal, and in two cases, the relapse was related to CPI re-start despite steroids.

**Figure 3:**
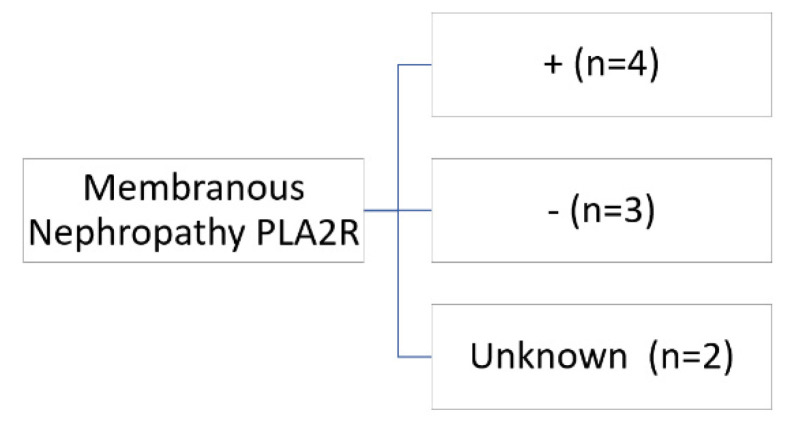
Antiphospholipase A2 antibodies in patients with membranous nephropathy.

A total of nine patients were diagnosed with membranous nephropathy. In only one patient, the renal diagnosis preceded the cancer diagnosis by five months. In two cases, membranous nephropathy was associated with drugs, and the anti PLA2R was negative: one with non-steroidal anti-inflammatory drugs (NSAIDs) and the other with anti-PD1. In the first case, the patient received treatment with empirical corticosteroids until the result of the kidney biopsy, which was withdrawn with subsequent complete remission. In the second case, immunotherapy (nivolumab) was removed, and the patient was treated for one month with corticosteroids at 0.5 mg/kg/day, and complete remission was achieved. The other seven patients were: four antiPLAR positive, one antiPLAR negative, and two unknown. In primary membranous nephropathy, the treatment administered in three cases was rituximab. (Figure 3, Table 6).

Half of the patients with thrombotic microangiopathy (8) received immunosuppressive treatments: eculizumab (*n* = 2), steroid (*n* = 1) and plasmapheresis (*n* = 1). Two of those required temporary dialysis despite the treatment. The renal damage was associated with gemcitabine in two patients and anti-VEGF in the rest.

All patients with extracapillary glomerulonephritis (*n* = 8) were treated with corticosteroids; four of them were treated with cyclophosphamide and three with rituximab.

Two patients with IgA nephritis were also treated with steroids. Table 7 summarizes the causal drugs of renal pathology in this series.

The mean follow-up was 15.2 months [5.7–31.4 (IQ 25–75)]. A total of 29 patients (18.9%) required kidney replacement therapy. There was an association between the presence of hematuria and the presence of nephrotic proteinuria with the requirement of renal replacement therapy (*p* = 0.003 and *p* = 0.006, respectively). Median creatinine at the end of the follow-up was 1.4 mg/dL [1.03–2.15 (IQ 25–75)], and 39.1% of patients had died. Of note, three months after the kidney biopsy, 33.8% of the patients had a serum creatinine > 1.5 mg/dL, while this percentage was 15.5% in baseline kidney function. At the end of the follow-up or before their death, 43.9% of the patients showed a serum creatinine > 1.5 mg/dL. The Cox survival analysis identified the presence of metastasis as a risk factor for mortality in our study (*p* = 0.006).

## 4. Discussion

As far as we know, this is the first multicenter study that analyzes the histologic and clinical patterns of kidney damage in patients with cancer submitted to a kidney biopsy over the last decade.

The main finding of this study is the evidence that the kidney pathology in patients with cancer is changing. Classically, membranous nephropathy was considered the paradigm of paraneoplastic kidney disease [18], and hemodynamic and urological were commonly detected as causes of acute kidney injury in this group of patients. However, our study demonstrates that almost 40% of patients with cancer who underwent a kidney biopsy in the last ten years had acute interstitial nephritis, mainly in patients under immunotherapy treatments. In general, we found that kidney disease was secondary to oncological treatment in 56% of patients and only related to cancer in 13%. In the first case, the drug responsible was withdrawn in 90% and subsequently reintroduced in 24%. These results could be due to the increased use of immunotherapy in oncological patients, being the first-line drugs for some tumors.

Acute interstitial nephritis is an entity secondary to checkpoint inhibitors or other classical drugs, which is easily treatable with steroids, and usually has a good prognosis if it is diagnosed early [8,9]. In our study, 21% of the patients suffered a recurrence in the following 3.6 months after discontinuation of the drug. Differentiating ATN from AIN in patients receiving immunotherapy or in those who had received it in previous lines of treatment is highly important to avoid temporary discontinuation of the drug or unnecessary corticosteroid treatment. Although biomarkers are being developed to help to differentiate these entities, such as IL-9 or TNF-alpha in urine [19,20], kidney biopsy remains the gold standard, providing prognostic information and discarding other less frequent entities. As suggested by the latest published works, kidney biopsies should be strongly considered if there are several alternatives that justify acute kidney failure [21]. If the patient is in a palliative situation and a renal event different from ATN is suspected, treatment with empirical corticosteroids would be a reasonable option.

IgA nephropathy was the second most common diagnosis, probably as a reflection of kidney disease causes in the general population [22] that could be extrapolated to oncologic patients. It is important to highlight that thrombotic microangiopathy was also present in more than 5% of patients, probably in relation to the use of anti-VEFG drugs. In addition, almost 10% of biopsies showed acute tubular necrosis, which is a well-known cause of AKI in these patients.

**Figure 4:**
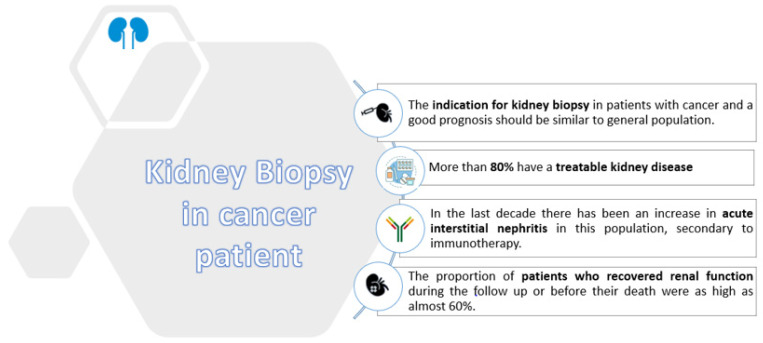
Kidney biopsy in cancer patients. Proposed beneficial results of performing a kidney biopsy in cancer patients with kidney disease.

When we analyzed all of the patients included in the study, we observed that 119 of 148 patients had potentially-treatable kidney disease (acute interstitial nephritis, IgA nephropathy, thrombotic microangiopathy, or another acute glomerulonephritis). Evaluation by a nephrologist with expertise in onconephrology could help find an etiological diagnosis of renal dysfunction in patients with cancer, avoiding diagnostic delays and improving renal prognosis. A better renal outcome would probably be linked to an increased number of future oncospecific treatments, generally limited by renal function (Figure 4). The proportion of patients who recovered renal function during the follow-up or before their death was as high as almost 60%, suggesting that kidney biopsy is useful for establishing therapeutic plans that lead to renal function improvement. Despite the good renal outcome, in our series, the mortality at the end of follow-up was 39%. However, this high mortality was mainly ascribed to the advanced cancer stage and not to the kidney disease “per se”.

As previously known, the prevalence of CKD in patients with cancer is increasing compared with the general population [23]. In our series, 15.5% of the patients presented a baseline creatinine > 1.5 mg/dL, which translates to an incidence of CKD in our population of at least 15.5%, and probably higher since we are not considering lower creatinine values or proteinuria. This result is similar to the Belgian BIRMA study, which showed that 18% of cancer patients presented glomerular filtration lower than 60 mL/min [23]. In concordance, Canter et al. also showed that 22% of patients with cancer presented CKD stage 3 in the USA [24].

It is interesting to mention that three months after the kidney biopsy, only 33.8% presented a creatinine > 1.5 mg/dL, possibly due to the pathological diagnosis of kidney disease and its appropriate targeted treatment, highlighting the importance of accurate nephrological evaluation in patients with cancer and de novo kidney disease.

The main reason for submitting a cancer patient to kidney biopsy was acute kidney failure [25,26,27]: classically, between 12% and 27% of oncologic patients develop AKI [28]. More recently, an increase in the incidence of AKI in cancer patients has been observed, from 18 to 52 cases per 1000 people-years from 2007 to 2014 [29]. This increase may be in part ascribed to the use of new therapies [7]. In the near future, the incidence of AKI in this population is reasonably expected to grow since today, the commonly used therapies, such as immunotherapy, are known to be linked to AKI episodes [11]. Furthermore, nowadays, patients submitted to oncological treatments are generally older and present with increasing comorbidities, such as type 2 diabetes or hypertension, which increase the risk of kidney failure.

Our study has several limitations. It is a retrospective study of a single medium-sized European country, so it may not apply to other regions with diverse populations and very different health systems. The information was obtained through the review of the clinical history, which could cause a lack of data. The strong point of the study, however, is that it is a multicenter study assessing the kidney biopsies performed in patients with cancer in the last ten years. Furthermore, this study is the first that analyzes only patients with cancer that underwent kidney biopsy, and it represents what is happening in real-world life medical practice.

## 5. Conclusions

Nowadays, there is a new trend in kidney disease and cancer patients. In our multicenter study, acute interstitial nephritis has been identified as the most common kidney disease histologic pattern in this population, followed by acute tubular necrosis and IgA nephropathy. Kidney biopsy in this group of patients provides valuable diagnostics and drives treatment, leading to a better renal prognosis in these patients. More studies are needed to increase the knowledge of the diagnosis, treatment, and prognosis of oncologic patients with kidney damage.

## Figures and Tables

**Table 1:** Baseline characteristics of the studied population.

Sample Size	148 Patients
Age	66.9 (SD ± 10.5)
Gender	64.2% men
Diabetes mellitus	29.7%
Arterial hypertension	62.2%
Systolic blood pressure	131 [120–146.2 (IQ 25–75)]
Non-steroidal anti-inflammatory drugs	12.2%
Renin-angiotensine aldosterone system blockade	62.8%
Cr prior to kidney biopsy (mg/dL)	1 [0.82–1.3 (IQ 25–75)]
Median Glomerular Filtration Rate by CKD-EPI (mL/min/1.73 m^2^)	61 [24.7–83.8 (IQ 25–75)]
Cr prior to kidney biopsy > 1.5 (mg/dL)	15.5%
Malignancies	
*-Lung*	29.1%
*-Melanoma*	10.8%
*-Abdominal*	25.0%
*-Genitourinary*	19.6%
*-Others*	15.5%
Oncological status	
*-In remission*	15.2%
*-Free from disease*	25.5%
*-Stable*	24.1%
*-In progression*	35.2%
Metastatic neoplasm at the time of kidney biopsy	49.7%

Quantitative variables are expressed as mean ± standard deviation (SD) or median [Interquartile (IQ) 25–75] depending on the normality of variables. Cr: Creatinine; SD: standard deviation.

**Table 2:** Clinical and laboratory characteristics at the time of renal biopsy.

Systolic blood pressure (mmHg)	131 [120–146.2 (IQ 25–75)]
Diastolic blood pressure (mmHg)	74 [68–81.2 (IQ 25–75)]
Median creatinine (mg/dL)	2.58 [1.81–4.1 (IQ 25–75)]
Median Glomerular Filtration Rate by CKD-EPI (mL/min/1.73 m^2^)	21.8 [12.7–34 (IQ 25–75)]
Urine protein/creatinine ratio (mg/g)	700 [256–2463 (IQ 25–75)]
Urine albuminuria/creatinine ratio (mg/g)	220 [46–1196 (IQ 25–75)]
Nephrotic range proteinuria	23%
Hematuria	53.1%
Eosinophiluria	10.8%
Leukocyturia	31.8%
ANCA (Neutrophil cytoplasmic antibodies)	6.8%
Antinuclear antibodies	13.5%
Low C3 and/or C4 serum levels	10.1%
C3 mg/dL (85–180)	127.2 (SD ± 36.8)
C4 mg/dL (10–40)	28.8 (SD ± 9.6)
pH	7.35 [7.29–7.39 (IQ 25–75)]
Bicarbonate	21.7 (SD ± 4.9)
K (mmol/L)	4.2 [4.0–4.7 (IQ 25–75)]
Na (mmol/L)	138.1 [136–140 (IQ 25–75)]
Ca (mg/dL)	8.9 [8.5–9.3 (IQ 25–75)]
Mg (mg/dL)	1.9 [1.7–2.2 (IQ 25–75)]
P (mg/dL)	4.1 [3.4–5.3 (IQ 25–75)]
Hb (g/dL)	10.7 (SD ± 2.2)
Platelets (×10^9^/L)	233.5 [189–312 (IQ 25–75)]
Haemolytic anemia and/or low platelet	6.8%

Quantitative variables are expressed as mean ± standard deviation (SD) or median [Interquartile (IQ) 25–75] depending on the normality of variables. C: Complement; K: potassium, Na: sodium, Ca: calcium, Mg: magnesium, P: phosphate, Hb: hemoglobin.

**Table 3:** Kidney biopsy diagnosis.

Pathological Diagnosis	N (%)
Acute interstitial nephritis	59 (39.9)
Acute tubular necrosis	13 (8.8)
IgA nephropathy	11 (7.4)
Membranous nephropathy	9 (6.1)
Thrombotic microangiopathy	8 (5.4)
Extracapillary glomerulonephritis	8 (5.4)
Amyloidosis	7 (4.7)
Nephroangiosclerosis	6 (4.1)
Membranoproliferative glomerulonephritis	4 (2.7)
Diabetic nephropathy	4 (2.7)
Vasculitis	3 (2)
Not classifiable	3 (2)
Focal and segmental glomerulosclerosis	3 (2)
Cancer cells infiltration	2 (1.4)
Sclerosing (Chronic kidney disease)	2 (1.4)
Normal	2 (1.4)
Chronic interstitial nephropathy	1 (0.7)
Endocapillary glomerulonephritis	1 (0.7)
Minimal change disease	1 (0.7)
Mesangial glomerulonephritis	1 (0.7)

**Table 4:** Kidney disease associated with cancer patients.

	Kidney Disease Secondary to Oncological Process–Paraneoplastic	Kidney Disease Secondary to Anticancer Drugs	Others
**Acute** **Kidney** **injury**	Membranoproliferative GN Amyloidosis Membranous nephropathy IgA Nephropathy Extracapillary GN TMA		Extracapillary GN
		AIN	IgA nephropathy
		TMA	AIN
		ATN	Membranoproliferative GN
		Chronic interstitial nephropathy	Vasculitis
		No classificable	Amyloidosis
		Extracapillary GN	ATN
		Extracapillary GN	No classificable
		Nephroangiosclerosis	Nephroangiosclerosis
**Chronic** **kidney** **disease**	No case	AIN	IgA nephropathy Focal and segmental glomerulosclerosis Nephroangiosclerosis Sclerosing
**Exacerbated chronic** **kidney** **disease**	Extracapillary GN	AIN	Amyloidosis Normal AIN
**Proteinuria**	Amyloidosis Membranous nephropathy	TMA Membranous nephropathy	Membranous nephropathy IgA nephropathy TMA Focal and segmental glomerulosclerosis Mesangial GN Nephroangiosclerosis Sclerosing Membranoproliferative GN

Acute interstitial nephritis (AIN); Thrombotic microangiopathy (TMA); Acute tubular necrosis (ATN).

**Table 5:** Causes of acute interstitial nephritis not secondary to immunotherapy.

**Antibiotics (*n* = 4)**	Meropenem, Ciprofloxacin, Vancomycin, Cefepime
**Non-immunotherapy antineoplastics (*n* = 13)**	MEK B-Raf inhibitor (*n* = 2), *Bacillus Calmette Guérin* (*n* = 3), Tyrosine Kinsae Inhibitor (*n* = 3), Anti-vascular endothelial growth factor (*n* = 4) and Carboxyplatin.
**Others (*n* = 3)**	Non-steroidal Anti-inflammatory drugs, Sarcoidosis, unknown cause.

**Table 6:** Clinical features membranous nephropathy.

Cancer	Time Kidney Biopsy	Clinical Features	PLA2R	Treatment	Renal Outcome	Cancer Outcome
**Sigma**	+2 years	Cr 1 mg/dL Prot 1.0893 mg/g	+	Rituximab	Progression to CKD	Progression
**G** **astrointestinal stromal tumor**	+1 week	Cr 0.8 mg/dL Prot 6 g/24 h	+	Neoplasm treatment	No remission	Death 5 months later
**Anal**	Previous 5 month	Cr 1.06 mg/dL Prot 2.630 mg/g	Unknown	Neoplasm treatment	Partial remission	Progression and death
**Bladder**	+3 month	Cr 1.2 mg/dL Prot 7.800 mg/g	+	Rituximab	Complete remission	Progression
**Bladder**	+4 years	Cr 3.34 mg/dL Prot 9.100 mg/g	−	Rituximab	Partial remission	Partial remission
**Intestinal**	+2 years	Cr 2.6 mg/dL Prot 8.034 mg/g	Unknown	Corticosteroids and cyclophosphamide	Partial remission	Progression
**Bladder**	+8 month	Cr 4.31 mg/dL Prot 13.840 mg/g	−	Empirical corticosteroids	Complete remission	
**Breast**	+5 month	Cr 0.7 mg/dL Prot 7886 mg/g	+	Rituximab	Relapse	Stable disease
**Melanoma**	+8 years	Cr 1.7 mg/dL Prot 1400 mg/g	−	Corticosteroids	Complete remission	

**Table 7:** Drugs associated with kidney disease.

Drug	Histopathological Diagnosis
Anti CTLA4 + PD1/PD-1/PD-L1	AIN, Extracapillary GN, Membranous nephropathy.
Pemetrexed	Chronic interstitial nephropathy
Emactuzumab	No classificable
Cisplatin	Nephroangioesclerosis
MEK/B-RAF	AIN
Anti VEGF	TMA
Oxiplatino	ATN
Alectinib	ATN
Capetitabine	ATN
Gemcitabine	TMA
Tyrosine kinase inhibitor	AIN
Bacillus Calmette Guérin	AIN

## Data Availability

Not applicable.

## Supplementary Materials

The following supporting information can be downloaded at: https://www.mdpi.com/article/10.3390/jcm11102915/s1, Table S1: List of patients contributed by each participating hospital. Figure S1: Histological diagnoses and treatments classified into two time periods: 2010 to 1016 and from 2017 to 2021. Figure S2: Opinion of the nephrologist.
