# Supplementary material for: Kidney Biopsy in Patients with Cancer along the Last Decade: A Multicenter Study
Source: J Clin Med. 2022 May 21;11(10):2915. doi: 10.3390/jcm11102915 (PMC9143132; doi:10.3390/jcm11102915)
Supplement: Supplementary file 1 [file jcm-11-02915-s001.zip › jcm-1677328-supplementary.pdf]

Supplementary material:

Supplementary Table S1	List of patients contributed by each participating hospital
Supplementary Figure S1	Histological diagnoses and treatments classified into two time periods: 2010 to 2016 and from 2017 to 2021
Supplementary Figure S2	Opinion of the nephrologist

Supplementary Table S1

List of patients contributed by each participating hospital:

**Hospital**	**N**
Vall d'Hebron University Hospital	48
Hospital Clinic Barcelona	20
Fundación Alcorcón	14
Bellvitge Hospital	13
Córdoba Hospital	13
Dr. Josep Trueta Hospital	12
Virgen Macarena Hospital	10
Clínico San Carlos Hospital	7
Gregorio Marañón Hospital	5
Hospital del Mar	3
Virgen del Rocio Hospsital	2
Navarra Hospital	1

Supplementary Figure S1

Histological diagnoses and treatments classified into two time periods: 2010 to 2016 and from

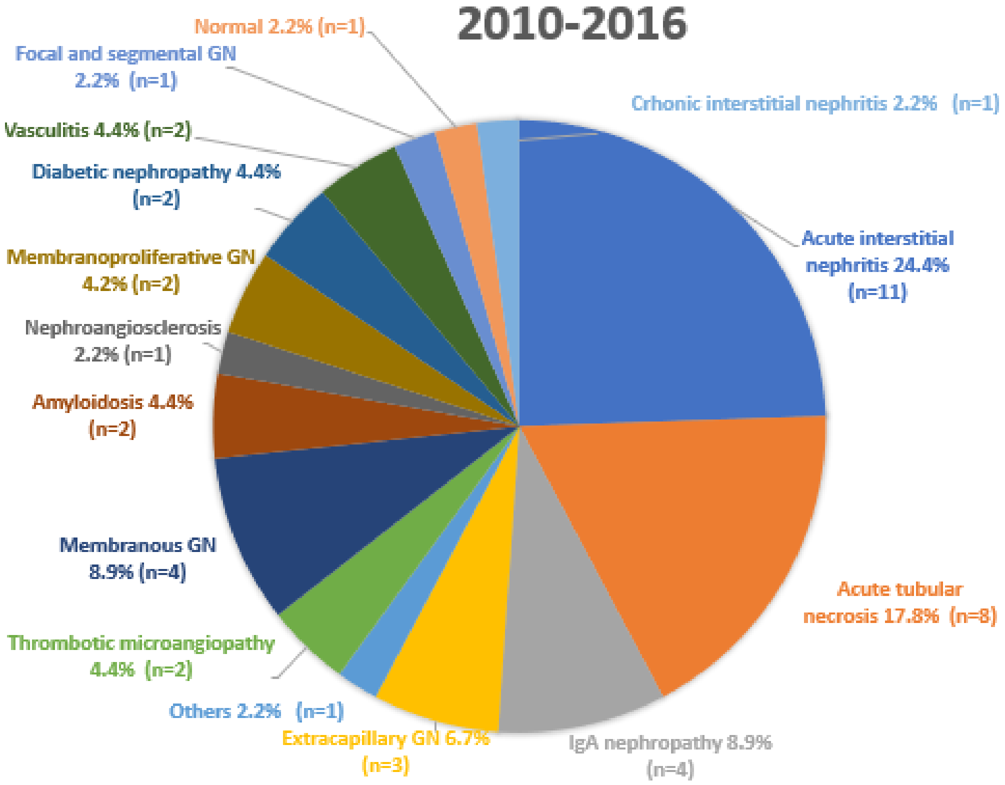

2017 to 2021.

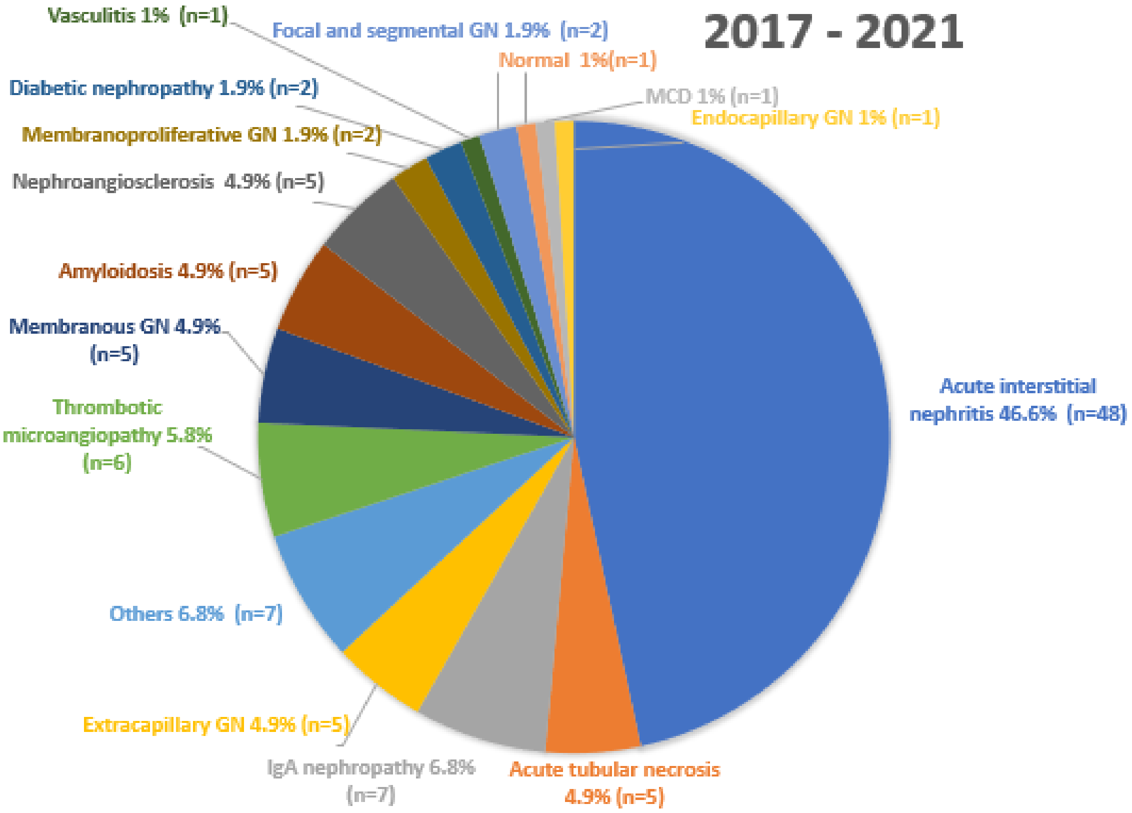

MCD: Minimal change disease

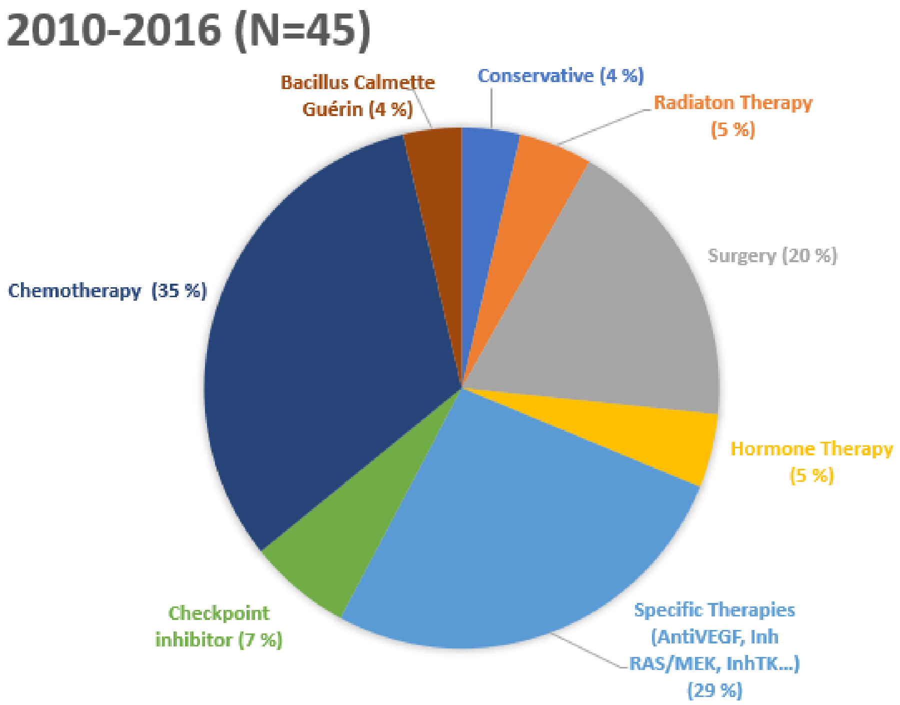

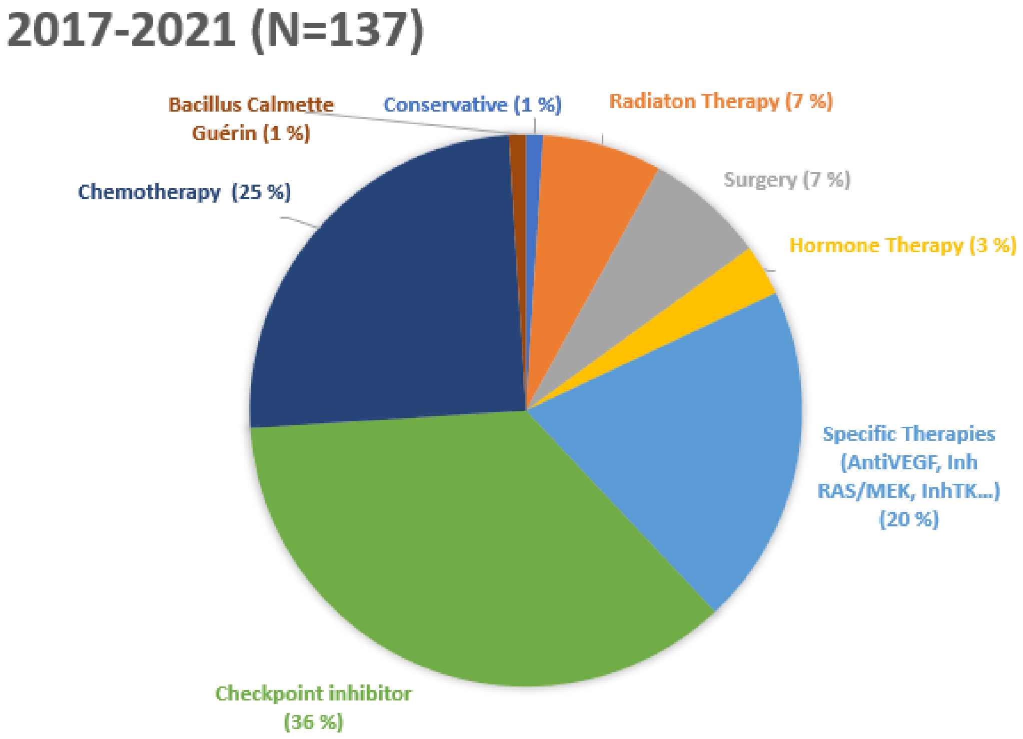

Supplementary Figure S2

Opinion of the nephrologist.

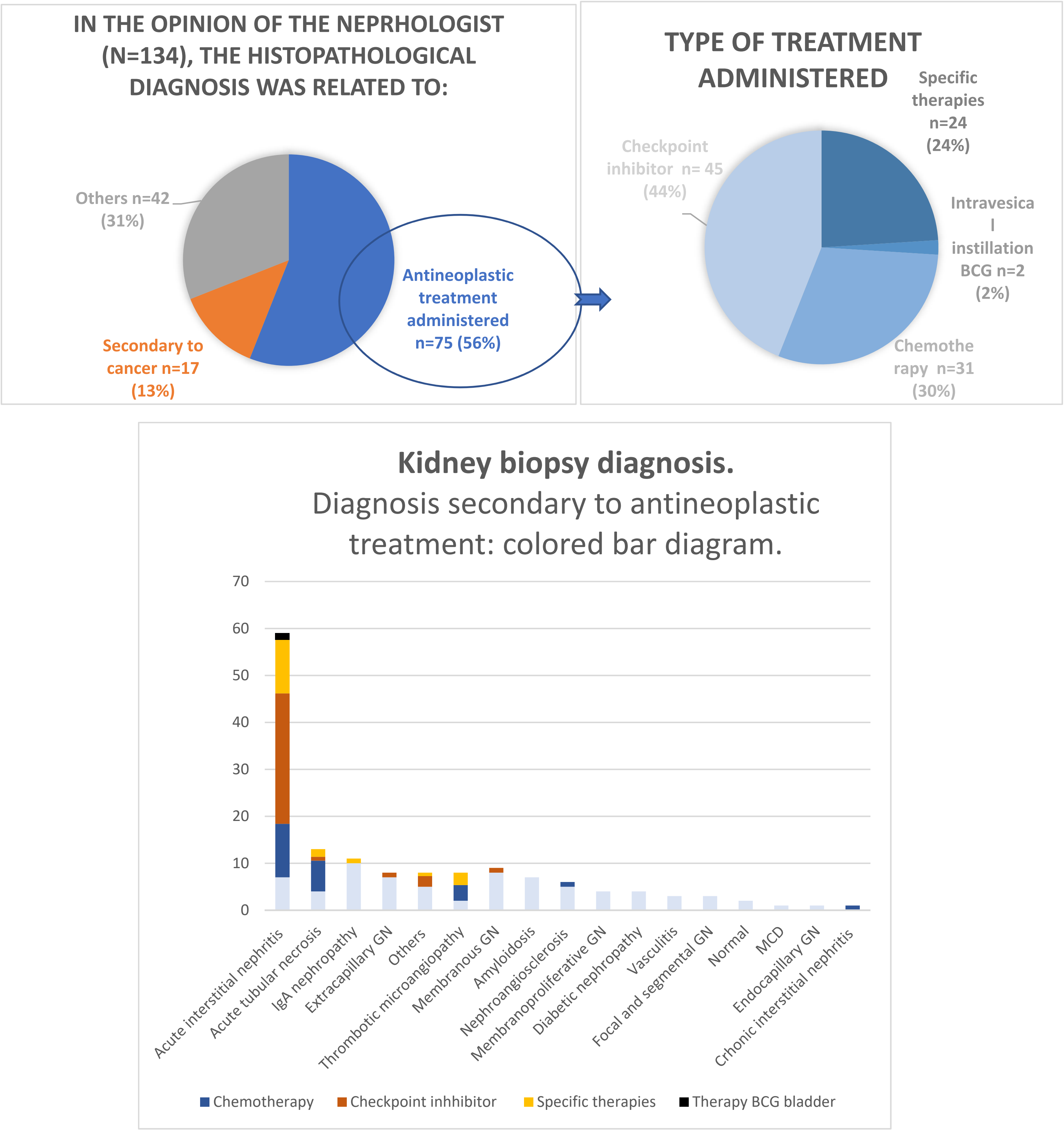

BCG: Bacillus Calmette Guérin.
